# Data in support of genome-wide identification of lineage-specific genes within *Caenorhabditis elegans*

**DOI:** 10.1016/j.dib.2015.07.032

**Published:** 2015-08-03

**Authors:** Kun Zhou, Beibei Huang, Ming Zou, Dandan Lu, Shunping He, Guoxiu Wang

**Affiliations:** a Hubei Key Laboratory of Genetic Regulation and Integrative Biology, Central China Normal University, Wuhan 430079, China; b The Key Laboratory of Aquatic Biodiversity and Conservation of the Chinese Academy of Sciences, Institute of Hydrobiology, Chinese Academy of Sciences, Wuhan 430072, China; c Huazhong Agriculture University, Wuhan 430070, China

## Abstract

Two sets of LSGs were identified using BLAST: *Caenorhabditis elegans* species-specific genes (SSGs, 1423), and *Caenorhabditis* genus-specific genes (GSGs, 4539). The data contained in this article show SSGs and GSGs have significant differences in evolution and that most of them were formed by gene duplication and integration of transposable elements (TEs). Subsequent observation of temporal expression and protein function presents that many SSGs and GSGs are expressed and that genes involved with sex determination, specific stress, immune response, and morphogenesis are most represented. The data are related to research article “Genome-wide identification of lineage-specific genes within *Caenorhabditis elegans*” in Journal of Genomics [1].

## Specifications table

**Table:** 

Subject area	*Biology*
More specific subject area	*Genomics*
Type of data	*Table, figure, image*
How data was acquired	*Download raw data online and analyze in silico using supercomputing blade systems*
Data format	*Analyzed*
Experimental factors	*Species of wild-type C. elegans N2 were cultured for a week, and subjected to a whole RNA extraction*
Experimental features	*RT-PCR experiments were conducted with 16 pairs of unique primers to amplify target sequences*
Data source location	*Wuhan, China*
Data accessibility	*Data is available with this article*

## Value of the data

**Table:** 

•	The data in our study shows the genetic features of SSGs and GSGs and that their expression profiles at different developmental stages.
•	The data of the origin analysis of SSGs and GSGs indicated that gene duplication and exaptation from TEs mainly generating these genes.
•	The data derived from protein function prediction in silico suggests SSGs and GSGs may be involved in specific stress, morphogenesis, and immune response, which indicates these genes might be relevant to some essential processes to adapt extreme environment.

## 1 Data, materials and methods

### 1.1 Sequence data

The proteomes of 58 vertebrate and 21 invertebrate species, excluding nematode species, were downloaded from Ensembl. The genomes and proteomes of nine *Caenorhabditis* species and 12 other nematode species were downloaded from WormBase. The UniProtKB protein data were downloaded from EBI (ftp://ftp.ebi.ac.uk/pub/databases/uniprot/knowledgebase/). All of the expression data (ESTs and mRNAs) of *Caenorhabditis elegans* were downloaded from UCSC (http://hgdownload.cse.ucsc.edu/downloads.html). The RNA-Seq data of *C. elegans* were obtained from EBI (http://www.ebi.ac.uk/ena/) and NCBI (http://www.ncbi.nlm.nih.gov/sra/).

### 1.2 Homolog search

**Fig. 1:**
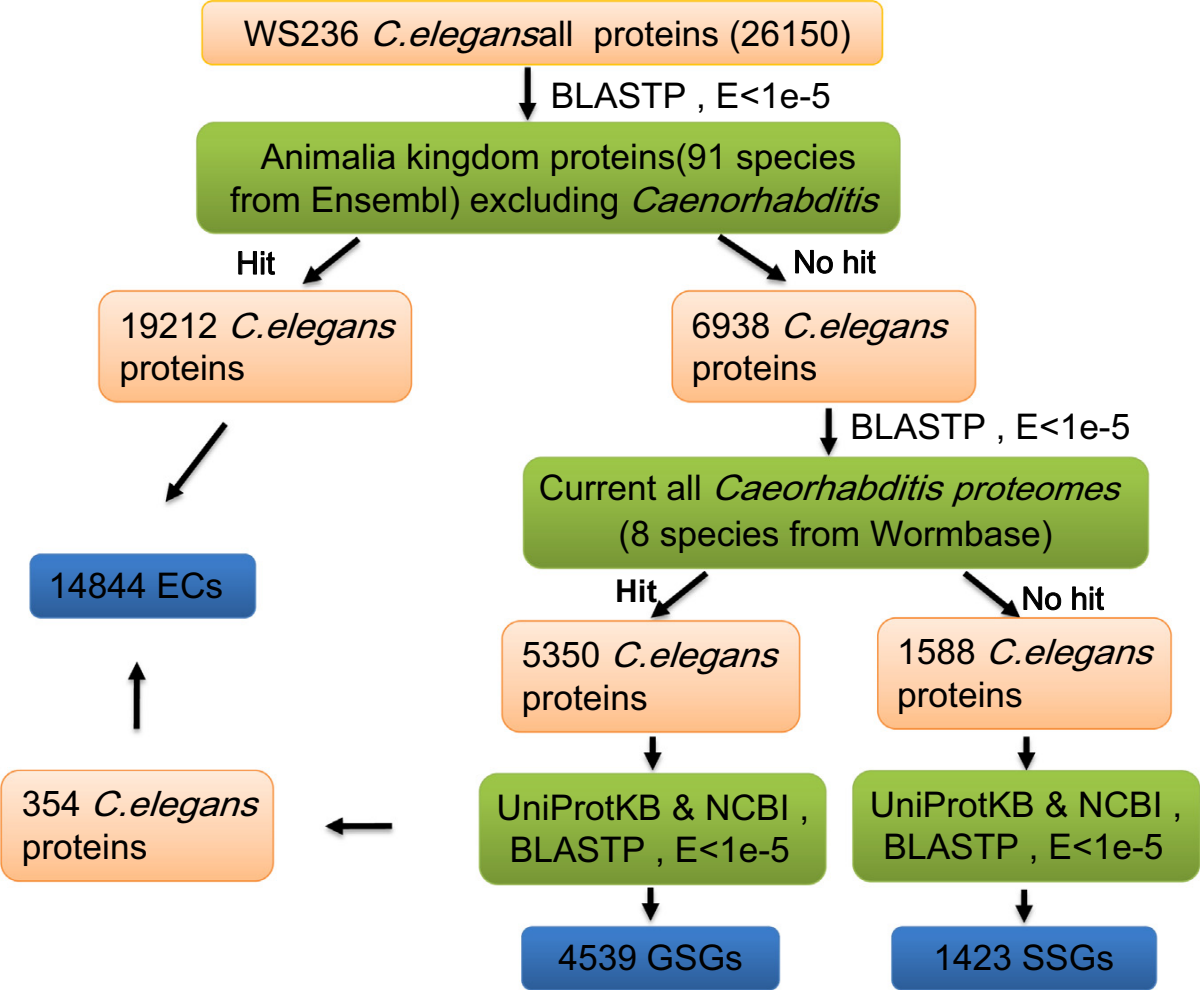
Procedure for identifying lineage-specific genes within *C. elegans*. BLASTP was primarily used in this pipeline with an *e*-value less than 1e−5. *C. elegans* proteins were marked in orange, the proteins of species excluding *C. elegans* in green, and the results of SSGs, GSGs, and ECs (evolutionarily conserved genes) in blue. “Hit” in this figure represents a *C. elegans* protein has BLAST hits in BLASTP, whereas “No hit” represents a *C. elegans* protein without any hit.

The two sets of lineage-specific genes (LSGs), namely SSGs and GSGs, within *C. elegans* were identified through a pipeline (Fig. 1) based on a homolog search using BLAST [2] with an *e*-value cutoff of 10^−5^ [3–6] and scoring matrices of Blossum62, Pam70, and Pam30. As the proteins are of different sizes and scored by different matrices, we divided 26,150 *C. elegans* proteins into three sets: a set of proteins that are less than 30 aa in length, a set between 30 and 70 aa, and a set more than 70 aa, for BLASTP analyses. The details of our result regarding these LSGs are shown in Supplementary File 1.

### 1.3 Characterization of SSGs, GSGs, and ECs

The genetic feature information for the LSGs was downloaded from Ensembl using BIOMART (http://www.ensembl.org/). We used Perl scripts to calculate the gene length, protein length, exon number, and GC content. In addition, we determined the significant differences between SSGs, CTSGs, and evolutionarily conserved genes (ECs) through one-way ANOVA. The gene length, protein length, exon number, and GC content are provided in Table 1.

### 1.4 Origin analysis of LSGs

We downloaded the information about the genomic positions of TE sequences from the UCSC Genome Browser, as well as that of paralogs from Ensembl, and compared their positions with that of SSGs and GSGs to identify the LSGs containing TE sequences and the LSGs having homologs. If a lineage-specific gene has a complete overlap with TEs, we consider it was generated by the mechanism of exaptation from TEs; if it completely overlaps with paralogs, we consider it was from gene duplication. The results are shown in Table 2.

However, for the retroposition, we referred to the previous study [7] and developed a sophisticated pipeline to screen the chimeric genes: we mapped the *C. elegans* protein sequences onto its genome using TBLASTN (*E*-value≤10^−3^) [8]. Then, we analyzed the TBLASTN results and aligned the best-selected matches with each protein using GENEWISE [9]. Only proteins having multiple exons were selected for subsequent analyses. In parallel, we extracted and merged adjacent homology matches (distance<40 bp) from the TBALSTN results, requiring the merged target sequences have significant similarity with the query on amino acid level (>30%) and more than 50 aa in length. After the above steps, similarity searches of the merged sequences against the multi-exon proteins were conducted using FASTA [10], and the closest matches were selected as candidate parental-retrogene proteins. Then we verified the absence of introns from these putative retrogenes with 10,000 bp flanking regions via GENEWISE, and screened out the sequences with scores over 35. We checked the match of each parental-retrogene sequence, observing at least two introns were contained in the matching part of the parental gene, and confirmed the alignment over 40%. After identification of the retrogenes, they were compared to the gene positions of the annotated genes on WormBase. We defined an annotated gene with a specific amount of overlap (coding sequence>50 bp) with a retrogene as a chimeric gene. If the overlap exceeded 90 bp, the chimeric genes were considered maybe as false positives derived from the parental genes or their flanking regions. Then we aligned the recruited coding sequences of retrogenes to their parental genes with extending 10,000 bp flanking regions to ensure the reliability of these chimeric genes. The complete information regarding retrogenes and chimeric genes identified is shown in Supplementary File 2.

### 1.5 Transcriptional analysis

Cleaned by SeqClean (https://sourceforge.net/projects/seqclean/), the 381,408 transcript sequences were mapped to the *C. elegans* genome using BLAT [11] with the default parameters. Then the following criteria were imposed to obtain high-quality and clearly mapped transcripts: mapping length≥150 bp, identity≥98%, coverage within mapping≥97%, and coverage within whole transcript≥75%. When a transcript was mapped to multiple genomic loci, we discarded the ambiguous ones (difference in BLAT scores<2%) and attained the best matches from the rest. Furthermore, the genomic positions of the LSGs were compared with the mapped positions of ESTs and full-length cDNAs. A gene that overlapped by more than 100 bp with the ESTs was considered likely to be expressed. We then determined the significant differences between SSGs, CTSGs, and ECs through a *t*-test.

**Fig. 2:**
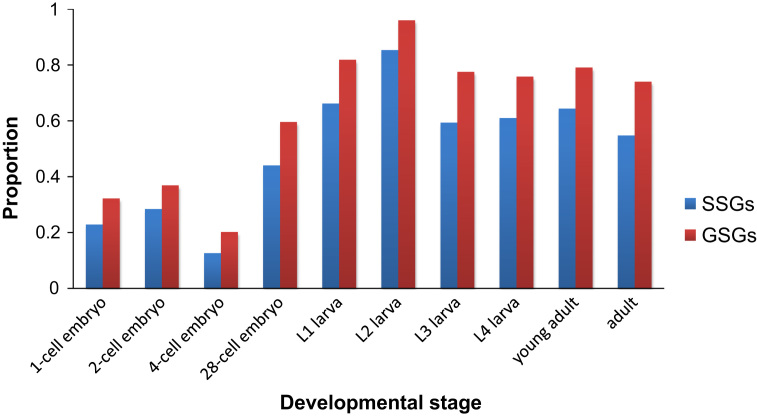
The proportion of LSGs expressed in different developmental stages. The vertical axis represents the proportion of genes with read supports, whereas, the abscissa axis represents the different developmental stages.

To further analyze the expression profile, we downloaded the RNA-Seq data from different embryo stages, including 1-cell, 2-cell, 4-cell, 28-cell, early, and late embryos with the accession codes SRX004864, SRX092477, SRX092371, SRX085219, SRX092479, and SRX004865, respectively [12]. Simultaneously, we downloaded information for the larva stages, including L1 larva, L2 larva, L3 larva, L4 larva, and L4 male larva with the accession codes SRX004867, SRX001872, SRX001875, SRX001874, and SRX004868, respectively [12]. Additionally, we downloaded the adult stages, including young adult, adult hermaphrodite, and adult male with the accession codes SRX001873, SRX191947, and SRX191950, respectively [12,13]. After downloading, the TopHat [14] and HTSeq [15] software programs were used to map all of the reads per time point independently back to the *C. elegans* genome and to then calculate the read number per gene. Moreover, the expression levels of LSGs were normalized to reads per kilobase per million mapped reads, which is known as the RPKM method. With the RPKM, we compared the number of expressed SSGs and GSGs at each developmental stage to observe their overall expression levels. After screening out the SSGs and GSGs expressed only at the larva and adult stages, we examined the possible motifs contained in their proteins using InterProScan. The complete information regarding SSGs and GSGs, including the RPKM values, is shown in Supplementary Files 3 and 4. To compare the number of expressed SSGs and GSGs, we calculated the proportion of expressed genes at each developmental stage and gain the result in Fig. 2. InterProScan results for the predicted functions are provided in Supplementary File 5.

### 1.6 Functional assignment and categorization of SSGs and GSGs

We downloaded the current developmental and functional descriptions of *C. elegans* from WormBase (http://www.wormbase.org/). Based on the descriptions, functional assignments of some SSGs and GSGs were obtained and clustered into different categories. A table summarizing the gene numbers of each class description of SSGs and GSGs is provided in Supplementary File 6. In addition, a table of the gene class descriptions of SSGs and GSGs is provided in Supplementary File 7. For the LSGs without annotations, we employed the ProtFun 2.2 server (http://www.cbs.dtu.dk/services/ProtFun/) to predict their cellular roles and gene ontology categories. The functions of LSGs predicted using ProtFun are provided in Supplementary File 8.

### 1.7 RT-PCR experiments

**Fig. 3:**
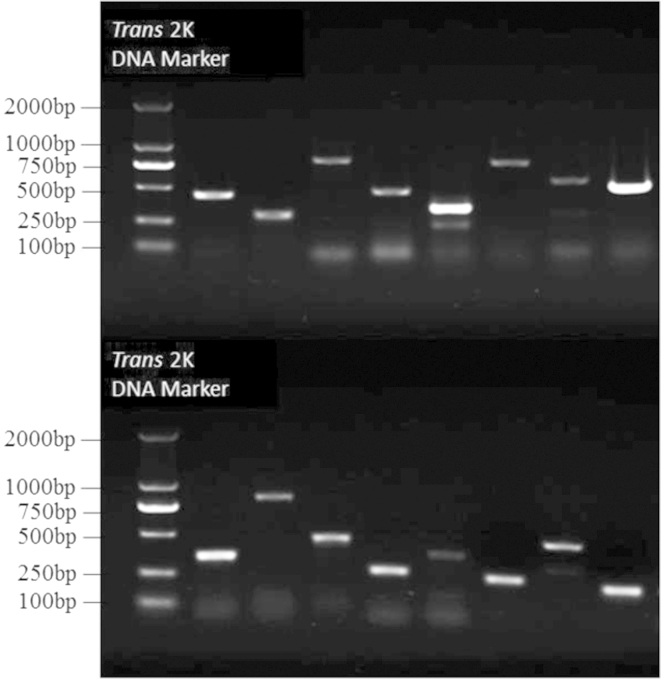
Results of RT-PCR. The first column represents the trans2k DNA Marker, and the other columns represent the 16 LSGs of WBGene00007393, WBGene00018297, WBGene00077563, WBGene00017986, WBGene00013778, WBGene00045297, WBGene00015229, WBGene00013713, WBGene00044080, WBGene00009308, WBGene00008603, WBGene00003764, WBGene00015821, WBGene00015597, WBGene00021289, and WBGene00002117, respectively.

The total RNA from a mixed-stage population of wild-type *C. elegans* N2, which was washed three times with an M9 buffer solution, was isolated using a Trizol Reagent kit (Invitrogen, Carlsbad, CA, USA). We then treated the RNA with Recombinant DNase I (TaKaRa, Japan) to eliminate genome pollution before reverse transcription. Simultaneously, we designed 16 pairs of unique primers to amplify the target sequences and then conducted RT-PCR experiments with a subset of the LSGs identified in our study. Gel image is shown in Fig. 3 and the primer information of RT-PCR is located in Supplementary File 9.

## Figures and Tables

**Table 1:** Characteristics of SSGs, GSGs, and ECs.

	**Gene size (nt), mean±SE**	**Exon number, mean±SE**	**Protein size (aa), mean±SE**	**GC content, mean±SE**	**Transcripts, %**
**SSGs**	1057.39±30.22	142.47±3.02	3.12±0.06	37.84±0.15	32.04
**GSGs**	1828.92±37.48	282.21±2.97	4.45±0.04	36.93±0.06	58.69
**ECs**	4744.42±41.00	500.43±3.80	7.45±0.04	37.07±0.03	80.97

**Table 2:** Categories of *C. elegans* LSGs.

Mechanism of Formation of *C. elegans* LSGs	*N*	
	SSGs	GSGs
Exaptation from TEs	374 (26.28%)	1588 (34.99%)
Gene duplication	355 (24.95%)	2527 (55.67%)
Exaptation from TEs and gene duplication	107 (7.52%)	887 (19.54%)
Total	622 (43.71%)	3228 (71.12%)

## Footnotes

Appendix A Supplementary data associated with this article can be found in the online version at doi:10.1016/j.dib.2015.07.032.
